# Association between *cagA* and *vacA* genotypes and pathogenesis in a *Helicobacter pylori* infected population from South-eastern Sweden

**DOI:** 10.1186/1471-2180-12-129

**Published:** 2012-07-02

**Authors:** Anneli Karlsson, Anna Ryberg, Marjan Nosouhi Dehnoei, Kurt Borch, Hans-Jürg Monstein

**Affiliations:** 1 Division of Surgery, Department of Clinical and Experimental Medicine, Faculty of Health Sciences, Linköping University, S-581 85, Linköping, Sweden; 2 Division of Clinical Microbiology, Department of Clinical and Experimental Medicine, Faculty of Health Sciences, Linköping University, Department of Clinical Microbiology, County Council of Östergötland, S-581 85, Linköping, Sweden

**Keywords:** Chronic gastritis, Atrophy, Intestinal metaplasia, Peptic ulcer, Duodenal ulcer, *CagA* EPIYA motif, *VacA* mosaic structure

## Abstract

**Background:**

Chronic gastritis, peptic ulcer disease, and gastric cancer have been shown to be related to infection with *Helicobacter pylori* (*H. pylori*)*.* Two major virulence factors of *H. pylori*, CagA and VacA, have been associated with these sequelae of the infection. In this study, total DNA was isolated from gastric biopsy specimens to assess the *cagA* and *vacA* genotypes.

**Results:**

Variations in *H. pylori cagA* EPIYA motifs and the mosaic structure of *vacA* s/m/i/d regions were analysed in 155 *H. pylori*-positive gastric biopsies from 71 individuals using PCR and sequencing. Analysis of a possible association between *cagA* and *vacA* genotypes and gastroduodenal pathogenesis was made by logistic regression analysis. We found that *H. pylori* strains with variation in the number of *cagA* EPIYA motif variants present in the same biopsy correlated with peptic ulcer, while occurrence of two or more EPIYA-C motifs was associated with atrophy in the gastric mucosa. No statistically significant relation between *vacA* genotypes and gastroduodenal pathogenesis was observed.

**Conclusions:**

The results of this study indicate that *cagA* genotypes may be important determinants in the development of gastroduodenal sequelae of *H. pylori* infection. In contrast to other studies, *vacA* genotypes were not related to disease progression or outcome. In order to fully understand the relations between *cagA*, *vacA* and gastroduodenal pathogenesis, the mechanisms by which CagA and VacA act and interact need to be further investigated.

## Background

*H. pylori* is a microaerophilic, spiral shaped Gram-negative bacterium that chronically infects the gastric mucosa [1]. It is recognised as a human pathogen associated with chronic gastritis [1], peptic ulcer [2] and gastric cancer [3], the development of which are related to the virulence factors cytotoxin associated antigen (CagA) [4,5] and *vacuolating cytotoxin A* (*vacA*) [6,7]. It has been reported that CagA and VacA polymorphisms are associated with distinct pathological features in *H. pylori* infected adults with gastrointestinal diseases [8-14].

**Figure 1:**
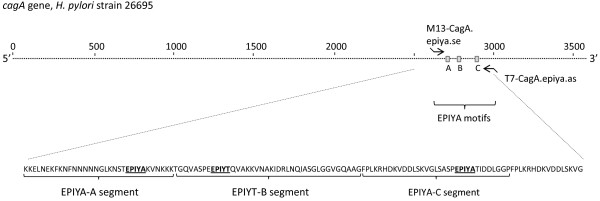
**A) Schematic illustration of the *H. pylori *26695 *cagA *gene.** M13-CagA.epiya.se and T7-CagA.epiya.as indicate the position of the primers used in PCR amplification. **B)** Amino acids flanking the EPIYA motifs present in EPIYA-A, EPIYA-B, and EPIYA-C segments of *H. pylori* 26695.

CagA has emerged as a major virulence factor for gastroduodenal disease severity, including an increased cancer risk [9,15]. CagA is injected into epithelial cells mediated by a type IV secretion system [4,16,17]. In the host cell, CagA localises to the inner surface of the plasma membrane and becomes phosphorylated on specific tyrosine residues within repeating penta amino acid Glu-Pro-Ile-Tyr-Ala (EPIYA) motifs present at the C-terminus of the protein [18-20]. This part of the protein is encoded by the variable 3’-region of the *cagA* gene [4,5,21,22] (Figure 1). Four different *cagA* EPIYA motifs have been defined according to the amino acid sequence that surrounds the EPIYA residues; EPIYA-A, -B, -C and -D [20,22-25]. CagA toxins nearly always possess EPIYA-A and EPIYA-B, followed by varying numbers of EPIYA-C in Western-type isolates [22]. In East Asian-type of clinical *H. pylori* isolates, EPIYA-A and -B are, on the other hand, commonly followed by an EPIYA-D motif [24,25]. It has been suggested that the considerable variation in number of repeating EPIYA-C motifs at the C-terminus of the protein may alter the biological activity of CagA in phosphorylation-dependent as well as phosphorylation-independent ways [20,26]. It was suggested that the number of *cagA* EPIYA-C motifs and the tyrosine phosphorylation status of CagA are important risk factors for gastric cancer among Western strains [27]. This is also supported by a higher risk of cancer development in strains with a high degree of phosphorylation [28].

**Figure 2:**
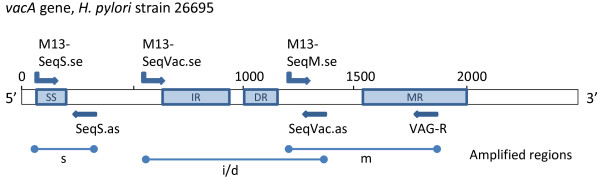
**Schematic illustration of the *H. pylori *26695 *vacA *gene.** The amplified signal-sequence region (SS), intermediate-region (IR), deletion-region (DR) and mid region (MR) and the primers used (Table 2) are indicated in blue. s, i/d, m indicate amplicons generated and sequenced.

VacA induces vacuolisation in gastric epithelial cells by attaching to the cell membrane [29]. It also induces apoptosis in these cells via the mitochondrial pathway [30-33]. Initially, DNA sequence analysis revealed that the VacA protein has a mosaic structure comprising allelic variations in the signal (s) and mid region (m) (Figure 2), each having two different alleles (s1/s2, m1/m2) with different biological activities [6,34]. The s and m regions have been associated with gastric cancer and the premalignant condition gastric mucosal atrophy [35,36]. Recently, it was proposed that an intermediate (i) region, located between the s and m regions (Figure 2), is associated with gastric cancer [27,37-40]. Similarly, a novel *vacA* gene deletion (d) region (Figure 2) has been described [36]. The d region is located between the *vacA* i and m regions, and involves a cleavage site crucial for the protein function and is associated with gastroduodenal diseases [36]. Amino-acid alterations in the repeated hydrophilic motif region (RHM), largely overlapping the d region of *vacA,* were previously shown not to be associated with any specific gastroduodenal disease [41].

*H. pylori cagA* and *vacA* gene polymorphisms are well studied and it is assumed that these polymorphisms, alone or in concert, are associated in *H. pylori* associated pathogenesis [9,10,13,42,43]. However, some studies have reported a lack of association between *H. pylori cagA* and *vacA* gene polymorphisms and the severity or progression of *H. pylori* associated diseases [25,44].

Statistical outcome is dependent on the population studied. We aimed to analyse a randomly selected population in South-eastern Sweden with regard to *H. pylori cagA* and *vacA* genotypes and sequelae using logistic regression analysis. By means of a previously described PCR-based strategy [45,46] we assessed variations of *cagA* EPIYA and *vacA* s/m/i/d mosaic structure present in *H. pylori* DNA isolated from 155 fresh frozen (−80°C) gastric biopsy specimens.

## Results

### Presence of *H. pylori* DNA in the gastric biopsy specimens

Using MDA-DNA and 16S rDNA variables V3 region pyrosequencing analysis, the presence of *H. pylori*-DNA in all 155 biopsy specimens was confirmed.

### Analysis of c*agA* EPIYA motifs

**Figure 3:**
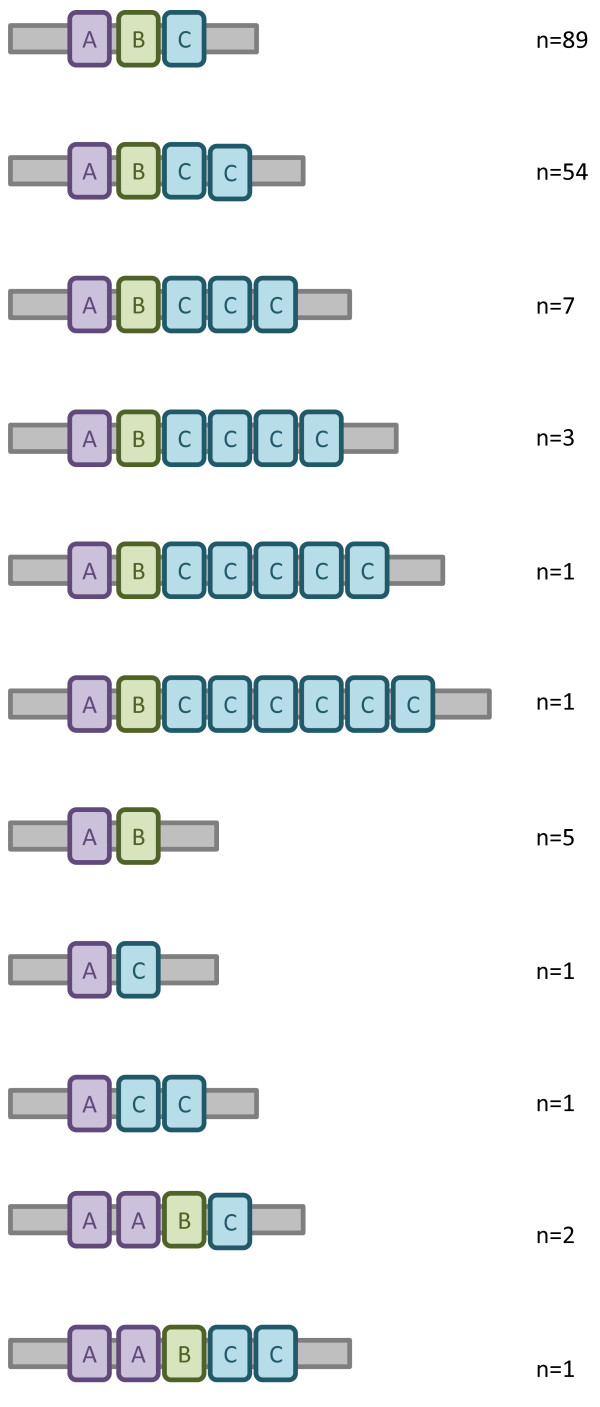
**Summary of the various *cagA *EPIYA motif combinations based on amplicon sequencing.** The large number of genotypes presented is due to biopsies having several motif combinations (multiple amplicons). For full information about EPIYA motifs in each biopsy, see Additional file 1. N = number of strains.

A total of 155 gastric biopsy specimens from 71 individuals were analysed for *cagA* EPIYA genotypes. In 92 biopsy specimens a single *cagA* amplicon was detected. DNA sequencing revealed the presence of different *cagA* EPIYA genotypes: EPIYA-AB in two, ABC in 56, ABCC in 29, and ABCCC, AC, ACC, AABC, AABCC in one biopsy each (Figure 3). In 37 biopsy specimens positive for the *cagA* EPIYA motif, two or more *cagA* amplicons were detected. Of these 37 specimens, the majority (19 specimens) were mixes between ABC and ABCC (Additional file 1). From 26 biopsy DNA samples, no *cagA* EPIYA motif amplicons could be generated.

Statistical analysis revealed that *H. pylori* strains with different number of *cagA* EPIYA motif variants present in the same biopsy was correlated to peptic ulcer development, OR = 2.77 (1.10-7.00). In the present study, peptic ulcer included four cases of duodenal ulcers, three pre-pyloric ulcers, two gastric ulcers and five cases of previously diagnosed ulcers of undefined origin (no data available).

Two or more *cagA* EPIYA-C motifs were associated with development of gastric atrophy, OR = 1.86 (1.05-3.30). In biopsies with mixed amplicons, the number of EPIYA-C was determined from the amplicon with the highest number of repeats. Gastritis was histologically classified according to the Sydney system, and atrophy of the gastric mucosa was graded from 1–3 (1 = mild, 2 = moderate, 3 = severe) [47]. For the purpose of the present study, moderate to severe atrophy of the gastric mucosa was classified as atrophic gastritis.

Statistical calculations were performed also in subgroups based on the location in the stomach (corpus, antrum). No differences were observed between the groups regarding their respective disease progression.

### Analysis of *cagE* and *cag*-PAI empty-site

To detect deletions of *cagA* within cagPAI, a region surrounding *cagA* (*cag*-PAI empty site) was amplified, as well as the *cagE* gene (also located within the *cag*-PAI). Amplification of *cagE* was successful in 114 of the biopsies. Of the remaining 41 biopsies, only 19 successfully amplified the *cag*-PAI empty site region, indicating the presence of mutated primer target sites or absence of *cagE*.

### Analysis of *vacA* s/i/d/m-region

Four regions of the *vacA* gene (s, m, i and d regions) were genotyped. PCR amplification and DNA sequence analysis in 155 *H. pylori* positive biopsy specimens revealed full information from all regions of *vacA* in 146 samples. Of the samples genotyped in the s region, the majority were of s1a (130) or s1b (19) genotype, while only three samples were s2 genotype. In the m region the distribution was more even, with 87 samples of m1 genotype and 64 samples of m2 genotype. DNA from 32 of the biopsies displayed a deletion of the d region (d2), while 115 isolates showed wild-type sequence (d1) in this region. The intermediate region is classified according to two different sequences, and may be of i1, i2, i1-i2 or i2-i1 genotype. In this material, 94 isolates were of i1 genotype, 24 isolates of i2 genotype and 31 isolates of i2-i1 genotype. None were of i1-i2 type.

**Figure 4:**
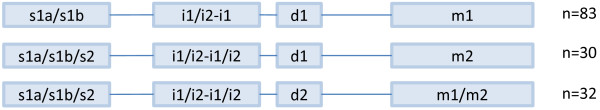
**Summary of the *vacA *gene mosaic combinations based on amplicon sequencing in 145 biopsies.** Genotypes in the remaining 14 biopsies could not be established. N = number of strains; s1/s2, signal-sequence type; i1/i2 = intermediate region type; d1/d2 = deletion type; m1/m2 = mid-region type.

The genotypes of these regions were divided into three groups (Figure 4); group 1 contained 83 isolates of s1-d1-m1 genotype (regardless of i region), group 2 contained 30 isolates that were of d1-m2 genotypes (regardless of the s and i regions), and group 3 contained 32 isolates that were of d2 genotype (regardless of all other regions). Isolates that were indeterminate in one or more regions (n = 9) were excluded from this compilation.

In group 3, there were two isolates (6%) derived from peptic ulcer patients, while in group 1 and 2 there were 20 isolates (24%) and eight isolates (27%), respectively, originating from ulcer patients. The lower frequency of peptic ulcer observed in *vacA* s1d1m1 genotype compared to other genotypes was not statistically significant. Eight biopsies from group 1 (10%) and two biopsies from group 2 (7%) were derived from patients with atrophic gastritis, while in group three there was no subject with atrophic gastritis (not statistically significant).

### Intraindividual variations of *cagA* EPIYA and *vacA* genotypes in corpus, antrum and duodenal bulb

In 51 of 71 individuals, biopsy specimens from all three locations of the stomach (corpus, antrum and duodenal bulb) were available for analysis. In 26 of these 51 subjects, the *cagA* and *vacA* genotypes were identical in all locations. Considering the remaining 25 subjects, 22 subjects differed with respect to the *cagA* EPIYA genotype, two with regard to the *vacA* (i) genotype, two considering the *vacA* (d) genotype and one with respect to the *vacA* (m) genotype, when comparing the locations for each subject (Additional file 1).

## Discussion

The results of several studies have indicated that there is an association between the *cagA* gene and gastric cancer [14,27,28,48]. There are also reports showing an association between the *vacA* gene and gastroduodenal sequelae (e.g. peptic ulcer, atrophic gastritis) of *H. pylori* infection [36,38-40].

Here we show that of the individuals with biopsies from all locationsns (corpus, antrum and duodenal bulb), 49% had different cagA EPIYA genotype between the three locations. There is a possibility that these individuals may have been infected with different strains on different occasions. However, it is perhaps more likely those *H. pylori* strains acquired genetic alterations in *cagA* after infection. Three recombination mechanisms have been detected in the *cagA* gene; homologous recombination between CM sequences, recombination between EPIYA sequences or between short similar sequences [49]. These recombination mechanisms, as well as mutations in the gene, may serve as a driving force for generating strain diversity in *H. pylori*, also called microevolution [50]. It is possible that infection with multiple *H. pylori* ancestral strains or alternatively, a single ancestral strain undergoing microevolution in the *cagA* gene giving rise to *H. pylori* subclones with different *cagA* EPIYA motif variants in the same biopsy specimen, may be more aggressive than a single ancestral strains acting alone. In an early study it has been suggested that the majority of Swedish clinical isolates of *H. pylori* from patients of higher age (>63 years old) represent single strain infections. However, in younger ages multiple strain infection may be more common [51]. Furthermore, it has been discussed that different subclones of each strain, some of which might be *cagA*-positive or -negative, may coexist, possibly colonising different areas of the stomach during different periods of life-long *H pylori* infection [51]. In this context, the aim of this study was to investigate a possible association between the presence of *H. pylori cagA* EPIYA motifs and disease outcome. We found an association between *H. pylori* DNA isolated from the same biopsy specimen and generating two or more *cagA* EPIYA motif variant amplicons and peptic ulcer OR = 2.77 (1.10-7.00).

Gastric atrophy was associated with two or more EPIYA-C motifs in the *cagA* gene of the biopsy (corpus and antrum only) *H. pylori* strains, OR = 1.86 (1.05-3.30). Previous studies have also found this correlation [14,27] and it has been suggested that a higher number of EPIYA-C motifs enables a higher degree of phosphorylation, and, hence, increases the risk of gastric cancer and gastric intestinal metaplasia [28]. One explanatory mechanism in this aspect may be the interaction of CagA with the protein ASPP2, which normally activates p53 to induce apoptosis. CagA inhibits ASPP2, leading to an increased cell survival and enhanced transformation of the cell [48].

Other studies have shown an association of gastric cancer and atrophy to the s1 genotype [35], the s1m1 genotype [36], or the i1 genotype [27,37-39]. In the present study, we detected a higher frequency of atrophy among the *vacA* s1d1m1 genotype than among other genotypes. However, none of these results were statistically significant, which could be due to small or unevenly distributed groups of samples (type II error).

Miernyk and co-workers observed an increased risk of developing peptic ulcer disease in s1m1 compared to s1m2/s2m2 *vacA* genotype [52]. Our study shows a tendency towards a similar association, although not statistically significant.

## Conclusions

In summary, *H. pylori* strains with variation in the number of *cagA* EPIYA motif variants present in the same biopsy were associated with the occurrence of peptic ulcer. Similarly, two or more EPIYA-C motifs were associated with atrophy in the gastric mucosa. No statistically significant association between *vacA* genotypes and gastroduodenal pathogenesis was observed.

## Methods

### Study subjects and tissue collection

Frozen (−80°C) gastric biopsy specimens from a gastroscopic re-screening study in a randomly selected cohort of the population of Linköping, County of Östergötland, South-eastern Sweden, were used [53]. The study was approved by the local ethics committee in Linköping, Sweden (Dnr. 98007) and conducted in accordance with the Helsinki declaration. In the clinical setting, *H. pylori* status was classified as positive when more than one of the following occurred: *H. pylori* identified by light microscopic examination; a positive urease test on fresh biopsy specimen; an elevated level of *H. pylori* IgG antibodies in serum. Microscopic examination was performed by a single experienced pathologist who was blinded to the other data. Kappa analysis of the blinded repeat evaluation of the Sydney system scores of the biopsy sections from the antrum and corpus has been described by Redéen and co- workers [47]. From this cohort, a total of 155 biopsy specimens (61 corpus, 57 antrum and 37 from the duodenal bulb) from 71 individuals fulfilling the criteria for presence of *H. pylori* infection, were selected and homogenized (Table 1). In 51 individuals, biopsies from both the corpus and antrum were available (Table 1).

**Table 1:** Number of individuals with biopsies from respective location

**Individuals with different biopsy combinations**^**1**^	**Corpus**	**Antrum**	**Duodenal bulb**
ABC	34	34	34
AC	14	14	
AB	-	2	2
BC	1	-	1
C	12	-	-
A	-	7	-
71	61	57	37

^1^A, Antrum; B, Duodenal bulb; C, Corpus.

DNA was isolated from the homogenized tissue using an automated nucleic extractor M48 and MagAttract DNA Mini M48 kit following the manufacturer’s instruction (Qiagen, Hilden, Germany). The isolated DNA was enriched by whole genome amplification by means of multiple displacement amplification (MDA), using an Illustra GenomiPhi V2 DNA kit (GE-Healthcare, Uppsala, Sweden) according to standard protocols.

### PCR amplification

Initially, the presence *H. pylori* DNA in the biopsy specimens were verified using 16S rDNA V3 region pyrosequencing analysis [54].

The *cagA* EPIYA motifs, located in the 3’-half of the *cagA* gene (Figure 1), were amplified using primer M13-CagA.EPIYA.SE and T7-CagA.EPIYA.AS (Figure 1; Table 2) The *cagE* gene and the *cagA* Pathogenicity Island (*cag*-PAI) empty site were amplified using primer M13-CagE.SE and CagE.AS, and primers M13(−21)_2.SE and T7_25.AS (Table 2), respectively.

**Table 2:** Primers used for PCR amplification in the study

**Amplicon**	**Primer**	**5' > 3'**^**1**^	**Size**	**Ref.**
VacA (s)	M13-SeqS.SE	*CGTTGTAAAACGACGGCCAGT*GACCCTTTGTGCAAAAATCGTT	381	[46]
	SeqS.AS	CCCARCCTCCATCAATCTT		
VacA (i + d)	M13-SeqVac.SE	*CGTTGTAAAACGACGGCCAGT*GAGCCAATTCAAYGGCAATTCT	803	[46]
	SeqVac.AS	CGCTTGATTGGACAGATTGA		
VacA (m)	M13-SeqM.SE	*CGTTGTAAAACGACGGCCAGT*GAAGTCRTTGATGGGCCTTTTG	717	[46]
	VAG-R	GCGTCAAAATAATTCCAAGG		
CagA/EPIYA	M13-cagA.EPIYA.SE	*TGTAAAACGACGGCCAGT*CCCTAGTCGGTAATGG(A/G)TT(A/G)TCT	580-830	[46]
	T7-cagA.EPIYA.AS	*TAATACGACTCACTATAGGGT*GTGGCTGTTAGTAGCGTAATTGTC		
Empty site CagA	M13(−21)_2.SE	*TGTAAAACGACGGCCAGT*ACATTTTGGCTAAATAAAC(A/G)CTG	375	[16]
	T7_25.AS	*TAATACGACTCACTATAGGGT*CATGCGAGCGGCGATGTG		[4]
CagE	M13-CagE.SE	*TGTAAAACGACGGCCAGT*GGGGGAATAGGTTGTTTGGT	385	[45]
	CagE.AS	GGATCACCCCATCATCTAAAAA		

^1^italic letters, universal tag sequence.

*VacA* s/i/d/m region subtyping was accomplished by three single PCR amplification assays. The signal-sequence (SS) region was amplified using primer M13-SeqS.se and SeqS.as; the intermediate and deletion region (IR and DR) using primer M13-SeqVac.se and SeqVac.as; the midregion (MR) using primer M13-SeqM.se and VAG-R (Figure 2; Table 2), respectively

Amplification conditions used were identical in all assays as described previously [45]. Prior to sequencing, amplicons were analysed by automated capillary gel electrophoresis using a QIAxcel system and a QIAxcel DNA High Resolution kit (Qiagen, Hilden, Germany).

### *cagA* EPIYA motif and *vacA* s/i/d/m-region sequence analysis

M13-tagged *cagA* EPIYA and *vacA* amplicons were sequenced using M13 uni (−21) sequencing primer and a customer sequencing service (Eurofins MWG Operon, Ebersberg, Germany). The obtained *cagA* and *vacA* sequences were aligned and compared with catalogued *H. pylori* 26695 [GenBank:AE000511, *H. pylori* J99 [GenBank:AE001439], *H. pylori* P12 [GeneBank:CP001217], *H. pylori* G27 [GenBank:CP001173], and *H. pylori* Shi470 [GeneBank:CP001072] sequences using the CLC DNA Workbench version 5.5 [55]. Sequences were retrieved from the NCBI nucleotide database [56]. *CagE* and *cag*-PAI (empty-site) amplicon sizes were analysed by capillary gel electrophoresis only.

### Statistical analysis

Binary logistic regression analysis of data was performed using Minitab 15 software. Statistical significance was assumed at *P* < 0.05. All statistical analyses presented here were significant according to Hosmer-Lemeshow (HL) goodness-of-fit test, with HL *p* values >0.05. In the logistic regression analysis and the GLM analysis, a 95% confidence interval including 1.0 was regarded as non-significant. Odds ratios with 95% confidence intervals (CI) were calculated to explore possible associations of individual genotypes to peptic ulcer or gastric atrophy. Age and sex were included as covariates. With regard to atrophy, data from duodenal biopsies were not included in the statistical analysis.

## Competing interests

The authors declare that they have no competing interests.

## Authors’ contributions

AK, AR, KB and HJM participated in the conception, design and data interpretation and drafting of the manuscript. AK, AR, MND performed the practical molecular biology procedures. KB collected and selected the biopsy specimens. All authors have been involved in drafting of the manuscript and approved the final version.

## Supplementary Material

Additional file 1 Results from *cagA *and *vacA *genotyping, including clinical data. Click here for file
