# Supplementary material for: Association between cagA and vacA genotypes and pathogenesis in a Helicobacter pylori infected population from South-eastern Sweden
Source: BMC Microbiol. 2012 Jul 2;12:129. doi: 10.1186/1471-2180-12-129 (PMC3520705; doi:10.1186/1471-2180-12-129)
Supplement: Additional file 1 — Results from cagA and vacA genotyping, including clinical data. [file 1471-2180-12-129-S1.pdf]

Additional file 1. Results from cagA and vacA genotyping, including clinical data.

Gastroscopy no.[1]		Age	*VacA* [2]					No. of *Hp* strains (*cagA*
			s	i	d	m	Group	
1	A	76	s1a	i2-i1	d1	m2	2	2
3	C	78	s1a	i2-i1	d1	m1	1	1
7	C	84	s1a	i2-i1	d2	m2	3	1
9	A	80	s1b	i1	d1	m2	2	1
9	B	80	s1b	i1	d1			1
9	C	80	s1b	i1	d1	m2	2	1
12	A	84	s1a	i1	d1	m2	2	1
12	C	84	s1a	i2-i1	d1	m2	2	1
13	A	81	s1a	i1	d1	m1	1	1
13	B	81	s1a	i1	d1	m1	1	1
14	C	82	s1b	i1	d1	m1	1	1
21	A	77	s1a	i2	d2	m2	3	1
26	A	87	s1a	i2	d2	m2	3	2
26	C	87	s1a	i2	d2	m2	3	2
27	A	82	s1a	i1	d1	m1	1	1
27	C	82	s1a	i1	d1	m1	1	1
28	A	80	s1a	i2	d2	m2	3	1
28	B	80	s1a	i2	d2	m2	3	1
28	C	80	s1a	i2	d2	m2	3	1
68	C	75	s1a	i2-i1	d2	m2	3	1
69	A	82	s1a	i1	d2	m2	3	1
69	B	82	s1a	i1	d2	m2	3	1
69	C	82	s1a	i1	d2	m2	3	1
71	A	71	s1a	i1	d1	m1	1	2
71	B	71	s1a	i1	d1	m1	1	1
71	C	71	s1a	i1	d1	m1	1	1
73	A	80	s1a	i1	d1	m1	1	1
73	C	80	s1a	i1	d1	m1	1	1
75	C	77	s1a					1
76	A	78	s1a	i2	d1	m2	2	1
76	C	78	s1a	i2	d1	m2	2	1
79	B	75	s1a			m2		1
79	C	75	s1a	i2	d2	m2	3	1
83	A	82	s1a	i2-i1	d2	m2	3	2
83	B	82	s1a	i2-i1	d2	m2	3	1
83	C	82	s1a	i2-i1	d2	m2	3	1
109	A	63	s1a	i2-i1	d1	m1	1	1
109	B	63	s1a	i2-i1	d1	m1	1	1
109	C	63	s1a	i2-i1	d1	m1	1	1
110	A	60	s1a	i2-i1	d1	m2	2	1
110	B	60	s1a	i2-i1	d1	m2	2	1
110	C	60	s1a	i2-i1	d1	m2	2	1
120	A	78	s1a	i1	d1	m1	1	1
120	B	78	s1a	i1	d1	m1	1	2
120	C	78	s1a	i1	d1	m1	1	1
121	A	75	s1a	i1	d1	m1	1	1
121	C	75	s1a	i1	d1	m1	1	2
125	A	80	s1a	i1	d1	m1	1	2
125	C	80	s1a	i1	d1	m1	1	3
127	C	78	s1a	i1	d1	m2	2	1
133	C	72	s1a	i1	d1	m1	1	1
142	A	60	s1a	i2	d2	m2	3	1
142	B	60	s1a					1
142	C	60	s1a	i2	d2	m2	3	1
144	A	77	s1a	i1	d1	m1	1	2
144	B	77	s1a	i1	d1	m1	1	2
144	C	77	s1a	i1	d1	m1	1	1
147	C	56	s1a	i2-i1	d1	m2	2	1

149	A	59	s1a	i2-i1	d1	m2	2	1
149	B	59	s1a	i2-i1	d1	m2	2	1
149	C	59	s1a	i2-i1	d1	m2	2	1
152	A	77	s1a	i1	d1	m1	1	3
152	B	77	s1a	i1	d1	m1	1	1
152	C	77	s1a	i1	d1	m1	1	2
154	A	49	s1a	i1	d1	m1	1	2
154	B	49	s1a	i1	d1	m1	1	1
154	C	49	s1a	i1	d1	m1	1	1
158	C	77	s1a	i1	d1	m1	1	1
162	A	75	s1a	i2-i1	d1	m1	1	2
162	B	75	s1a	i2-i1	d1	m1	1	2
169	A	72	s1a	i1	d1	m1	1	1
169	B	72	s1a	i1	d1	m1	1	1
169	C	72	s1a	i1	d1	m1	1	1
174	A	67	s1a	i1	d1	m2	2	2
178	A	62	s1a	i1	d1	m1	1	1
178	B	62	s1a			m1	1	1
178	C	62	s1a	i1	d1	m1	1	1
187	A	73	s1a	i1	d1	m1	1	1
187	B	73	s1a	i1	d1	m1	1	1
187	C	73	s1a	i1	d1	m1	1	1
193	C	71	s1b	i1	d1	m1	1	2
196	A	69	s1b	i1	d2	m2	3	1
196	B	69	s1b					1
196	C	69	s1b	i1	d2	m2	3	1
201	A	63	s1a	i1	d1	m1	1	1
201	B	63	s1a	i1	d1	m1	1	2
201	C	63	s1a	i1	d1	m1	1	2
207	A	70	s1a	i2-i1	d1	m2	2	1
207	B	70	s1a	i2-i1		m2		1
207	C	70	s1a	i2-i1	d1	m2	2	1
208	A	74	s1a	i2	d2	m2	3	1
208	C	74	s1a	i2	d2	m2	3	1
214	A	71	s1b	i2-i1	d1	m1	1	1
214	B	71	s1b	i2-i1		m1	1	1
214	C	71	s1b	i2-i1	d1	m1	1	2
220	A	66	s1a	i2	d1	m2	2	1
222	A	67	s1a	i1	d1	m2	2	1
222	C	67	s1a	i1	d1	m2	2	1
227	A	56	s2	i2	d2	m2	3	1
227	B	56	s2	i2	d2	m2	3	1
227	C	56	s2	i2	d1	m2	2	1
228	A	57	s1a	i2-i1	d1	m2	2	1
228	B	57	s1a	i2-i1	d1	m2	2	1
228	C	57	s1a	i2-i1	d1	m2	2	2
238	A	66	s1b	i2	d2	m2	3	1
238	C	66	s1b	i2	d2	m2	3	1
242	A	57	s1a	i1	d1	m2	2	2
242	B	57	s1a	i1		m2		1
242	C	57	s1a	i1	d1	m2	2	2
246	A	65	s1a	i1	d1	m1	1	1
246	B	65	s1a	i1	d1	m1	1	1
246	C	65	s1a	i1	d1	m1	1	1
253	A	52	s1a	i1	d1	m1	1	1
253	C	52	s1a	i1	d1	m1	1	1
264	A	65	s1a	i1	d1	m1	1	2
264	B	65	s1a	i1	d1	m1	1	1
264	C	65	s1a	i1	d1	m1	1	1
267	A	61	s1a	i1	d2	m1	3	1
267	C	61	s1a	i2	d2	m2	3	1
273	A	52	s1a	i1	d1	m1	1	1

273	B	52	s1a	i1	d1	m1	1	1
273	C	52	s1a	i1	d1	m1	1	1
275	A	51		i1	d1	m1		1
275	B	51		i1	d1	m1		1
275	C	51		i1	d1	m1		2
281	A	73	s1a	i1	d1	m1	1	2
281	B	73	s1a	i1	d1	m1	1	2
281	C	73	s1a	i1	d1	m1	1	1
290	A	48	s1b	i1	d1	m1	1	1
290	C	48	s1b	i1	d1	m1	1	1
292	A	59	s1a	i1	d1	m1	1	1
309	A	70	s1a	i1	d1	m1	1	2
309	B	70	s1a	i1	d1	m1	1	1
309	C	70	s1a	i1	d1	m1	1	1
310	A	56	s1a	i1	d1	m1	1	1
310	B	56	s1a	i1	d1	m1	1	1
310	C	56	s1a	i1	d1	m1	1	5
324	A	62	s1a	i1	d1	m1	1	1
328	A	56	s1a	i2	d2	m2	3	1
329	C	65	s1a	i1	d1	m2	2	1
335	A	66	s1a	i1	d1	m1	1	1
335	C	66	s1a	i1	d1	m1	1	1
346	A	58	s1a	i1	d1	m1	1	2
346	B	58	s1a	i1	d1	m1	1	1
346	C	58	s1a	i1	d1	m1	1	2
350	A	65	s1a	i1	d1	m1	1	2
350	B	65	s1a	i1	d1	m1	1	2
350	C	65	s1a	i1	d1	m1	1	2
351	A	69	s1b	i2	d2	m2	3	1
351	B	69	s1b	i2	d2	m2	3	1
351	C	69	s1b	i2	d2	m2	3	1
352	A	68	s1a	i2-i1	d1	m2	2	1
352	B	68	s1a	i2-i1	d2	m2	3	1
352	C	68	s1a	i2-i1	d1	m2	2	2
372	C	78	s1b	i1	d1	m1	1	2

[1] A, antrum; B, bulbus duodeni; C, corpus.

[2] Missing data is due to unreadable sequences probably caused by mixed strains in the sample

[3] ES, cagA empty site

CagA		Peptic ulcer	Inflammation grade 0-3	Atrophy, grade 0-3	Intestinal metaplasia
EPIYA genotype[3]	Maximum no. of EPIYA-C				
ABC+ABCC	2	1	2	1	1
ABC	1	0	2		0
ABCC	2	0	2	1	0
ABCC	2	0	2	0	0
ABCC	2	0	0		
ABCC	2	0	2	1	0
ES	0	0	2	0	0
ES	0	0	2	0	0
ES	0	0	2	1	0
ES	0	0	0		
ABCC	2	0	3	2	1
AC	1	0	2	0	1
ABC+ABCC	2	0	2	0	0
ABC+ABCC	2	0	1	0	0
ABC	1	0	1	0	0
ABC	1	0	2	1	0
ABC	1	0	2	1	0
ABC	1	0	2		
ABC	1	0	1	0	0
ES	0	0	1	1	1
ABC	1	0	1	0	0
ABC	1	0	0		
ABC	1	0	1	0	0
ABC+ABCC	2	0	1	1	1
ABCC	2	0	0		
ABCC	2	0	1	0	0
ABC	1	0	1	1	0
ABC	1	0	2	1	0
ES	0	0	1	0	0
ABCC	2	0	0	0	0
ABCC	2	0	1	3	3
ABC	1	0	0		
ABC	1	0	2	0	0
ABC+ES	1	0	1	0	1
ABC	1	0	0		
ABC	1	0	1	0	0
ABC	1	0	1	0	0
ABC	1	0	0		
ABC	1	0	2	2	1
ABC	1	0	2	0	0
ABC	1	0	0		
ABCC	2	0	1	0	0
ABC	1	0	2	0	0
ABC+ABCC	2	0	0		
ABCC	2	0	1	0	0
ABC	1	0	2	0	0
ABC+ABCCC	3	0	3	2	0
ABCC+ABCCC	3	1	2	3	3
ABC+ABCC+ABCCCC	4	1	3	2	1
ABC	1	0	2	0	0
ABCC	2	0	0	0	0
ABC	1	0	2	0	1
ES	0	0	0		
ABC	1	0	1	0	0
AB+ABC	1	0	2	1	1
AB+ABC	1	0	2		
ABC	1	0	1	0	0
ABC	1	1			

ABCC	2	0	2	2	2
ABCC	2	0	0		
ABCC	2	0	1	0	0
ABC+ABCC+ABCCC	3	0	2	1	1
ABCC	2	0	0		
ABC+ABCC	2	0	2	0	0
ABC+ES	1	1	0	0	0
ABC	1	1	0		
ABCC	2	1	1	0	0
ABCC	2	1	1	0	0
ABC+ABCC	2	0	2	1	0
ABC+ABCC	2	0	0		
ES	0	0	1	0	0
ES	0	0	0		
ES	0	0	1	0	0
ABC+ES	1	0	1	0	0
ABC	1	0	2	0	0
ABC	1	0	0		
ABC	1	0	1	0	0
ES	0	0	1	1	0
ES	0	0	0		
ES	0	0	2	2	2
ABC+ABCC	2	0	1	0	0
ABC	1	0	2	1	0
ABC	1	0	1		
ABC	1	0	2	1	0
ABC	1	1	2	0	0
ABC+ABCC	2	1	0		
ABC+ABCC	2	1	1	0	0
ABC	1	0	1	0	0
ABC	1	0	0		
ABC	1	0	1	0	0
ES	0	0	2	0	0
ES	0	0	2	0	0
ABCC	2	0	1	0	0
ABCC	2	0	0		
ABCC+ABCCC	3	0	1	0	0
ES	0	0	2	0	0
AB	0	1	1	0	0
AB	0	1	1	0	0
ES	0	0	1	0	0
ES	0	0	0		
ES	0	0	1	0	0
ABC	1	1	1	0	0
ABC	1	1	0		
ABC+ABCC	2	1	1	0	0
ES	0	0	1	0	0
ES	0	0	1	0	0
ABC+ABCC	2	0	2	0	0
ABCCC	3	0	0		
ABCC+ABCCCC	4	0	1	0	0
ES	0	0	2	0	0
ES	0	0	0		
ES	0	0	1	0	0
ES	0	1	1	0	0
ES	0	1	1	0	0
ABC+ABCC	2	1	1	0	0
ABCC	2	1	0		
ABCC	2	1	1	0	0
ABC	1	1	2	1	0
ABC	1	1	1	0	0
ABC	1	0	2	1	1

ABCC	2	0	2		
ABC	1	0	1	0	0
ABC	1	0	2	0	0
ABC	1	0	0		
ABC+ABCCC	3	0	1	0	0
ABC+ABCC	2	1	2	1	1
ABC+ABCC	2	1	0		
ABC	1	1	1	0	0
ABCC	2	0	2	0	1
ABCC	2	0	1	0	0
ABC	1	0	2	0	0
AABC+ES	1	0	1	0	1
AABC	1	0			
AABCC	2	0	1	0	0
ABC	1	1	1	1	1
ABC	1	1	0		
ABC--ABCCCCCC	6	1	0	0	0
ABC	1	0	1	0	1
ABC	1	0	1	0	0
ACC	2	1	1	0	0
ABCC	2	0	1	0	0
ABCC	2	0	1	0	0
AB+ABC	1	0	2	0	0
ABC	1	0	0		
ABC+ABCC	2	0	1	0	0
ABC+ES	1	0	2	3	3
ABC+ES	1	0	0		
ABC+ES	1	0	1	0	0
ABC	1	0	2	0	0
ABC	1	0	0		
ABC	1	0	1	0	0
ABCC	2	0	2	0	0
ABCC	2	0	0		
ABC+ABCC	2	0	2	0	0
ABC+ABCC	2	0	1	2	0

Pseudopyloral metaplasia (corpus)	Activity
0	2
1	1
0	1
0	1
	2
0	2
	3
	1
0	3
0	2
0	1
	1
1	1
	1
0	2
	0
0	1
0	1
	1
0	1
	1
0	1
	1
0	1
1	0
	1
0	
	2
0	1
	1
1	1
	2
0	1
	0
	1
	1
1	2
	2
2	2
	3
0	1
0	0
0	2
	0
0	2
	1

0	1
	0
0	2
	2
0	0
	0
0	1
0	1
0	1
	0
0	1
0	1
	0
0	0
	0
0	1
	2
	1
0	1
	1
0	1
	1
0	2
	1
0	1
	0
0	1
0	2
	0
0	1
	0
0	1
	1
0	1
	0
0	2
	1
0	1
	1
0	1
	0
0	1
	1
0	1
	0
0	1

	0
	1
	0
0	1
	1
0	1
	0
0	1
0	1
	0
0	1
	0
0	1
0	1
1	1
0	1
	1
0	1
	1
0	1
	1
0	2
	1
0	2
	2
1	1
